# Antimicrobial therapy using vancomycin and therapeutic drug monitoring (TDM) in patient with bacteremia caused by *Arthrobacter woluwensis*:a case report

**DOI:** 10.1186/s40780-025-00430-9

**Published:** 2025-03-20

**Authors:** Junichiro Date, Takayoshi Takeno, Shiro Watanabe, Akira Oda

**Affiliations:** 1 Department of Pharmacy, Shinrakuen Hospital, 3-3-11 Shindori-Minami, Nishi-ku, Niigata, 950-2087 Japan; 2 Education and Research Center for Clinical Pharmacy, Faculty of Pharmacy, Niigata University of Pharmacy and Medical and Life Sciences, 265-1, Higashijima, Akiha-ku, Niigata, 956-8603 Japan; 3 Department of Gastroenterology, Shinrakuen Hospital, 3-3-11 Shindori-Minami, Nishi-ku, Niigata, 950-2087 Japan

**Keywords:** *Arthrobacter woluwensis*, Vancomycin, Therapeutic drug monitoring (TDM), Gram-positive rod

## Abstract

**Background:**

*Arthrobacter woluwensis* (*A.woluwensis*) is gram-positive rod that is endemic to natural environments such as soil, but reports of infections caused by this species are limited, and effective treatment methods have not been established.

**Case presentation:**

An 89-year-old man was hospitalized for dysmobility due to anorexia and was started on peripheral intravenous nutrition. He had a fever of 39.5 °C, shivering, and hypotension. Gram-positive rods were detected in two sets of blood cultures. The treatment using vancomycin (VCM) was started due to suspicion of catheter-related bloodstream infection. The organism was identified as *A.woluwensis* by MALDI-TOF MS. The MIC_50_ for VCM was 2 µg/mL. Treatment was continued with the goal of achieving an area under the concentration-time curve (AUC) of ≥ 400 µg·h/mL, which is an indicator of the efficacy and safety of VCM in treating MRSA infection. The fever resolved after starting treatment, and the patient’s condition stabilized. Further blood cultures became negative, a transthoracic echocardiogram confirmed the exclusion of infective endocarditis, and the treatment was completed after 14 days.

**Conclusions:**

This is the first case report using VCM for the treatment and therapeutic drug monitoring (TDM) of *A. woluwensis* bacteremia. Our results will provide useful information for appropriate infection treatment.

## Background

*Arthrobacter woluwensis* (*A. woluwensis*) is a gram-positive rod that is endemic to natural environments such as soil [1]. Bloodstream infection caused by *A. woluwensis* have been reported, mainly catheter-related bloodstream infections and infective endocarditis in immunocompromised patients [2–8]. In these reports, the organism is susceptible to penicillin and anti-methicillin-resistant *Staphylococcus aureus* (MRSA) drugs, and antimicrobial therapy is mainly based on susceptibility, but the number of cases is limited and effective treatment methods have not been established.

Area under the concentration-time curve (AUC)-based dosing design is recommended to ensure the clinical efficacy and safety of vancomycin (VCM) in MRSA infection [9]. However, clinical investigation of therapeutic drug monitoring (TDM) in non-MRSA infections is lacking, and clinical efficacy is considered priority in TDM.

In this report, we describe a case of bloodstream infection caused by *A. woluwensis* that was successfully treated with vancomycin and a dosing design based on blood concentration.

## Case presentation

An 89-year-old Japanese man. He had medical records of hypertension and dyslipidemia. He was admitted for examination and treatment for dysmobility and dehydration due to decreased appetite. On the day of admission, peripheral intravenous nutrition was initiated with an amino acids, glucose, and electrolytes, vitamins solution (Pareplus^®^ 1000mL, Yoshindo) and an electrolyte solution (YDSolita-T1^®^ 500mL, Yoshindo) for him. These solutions were continuously infused over 24 h. For the purpose of this report, the day of admission is defined as day 1. Upper gastrointestinal endoscopy was performed, and he was diagnosed with advanced esophageal cancer and gastric ulcers. He was in a state of delirium that resulted in self-extraction of the peripheral venous catheter on day 2 and 3. Accordingly, from day 3, the infusion was changed to a 12-hour infusion within daytime, with daily replacement until day 8, when the patient’s symptoms subsided. Punctures were performed every day, at different sites on either side of the arm.

**Fig. 1:**
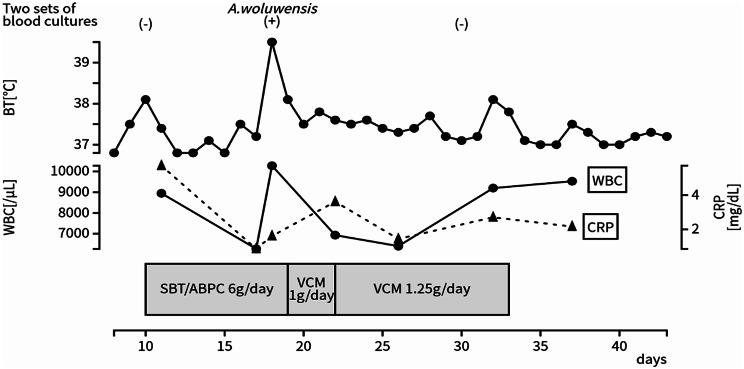
Clinical course of this case. *A.woluwensis*,* Arthrobacter woluwensis*; BT, body temperature; WBC, white blood cell; CRP, C-reactive protein; SBT/ABPC, sulbactam/ampicillin; VCM, vancomycin;

**Fig. 2:**
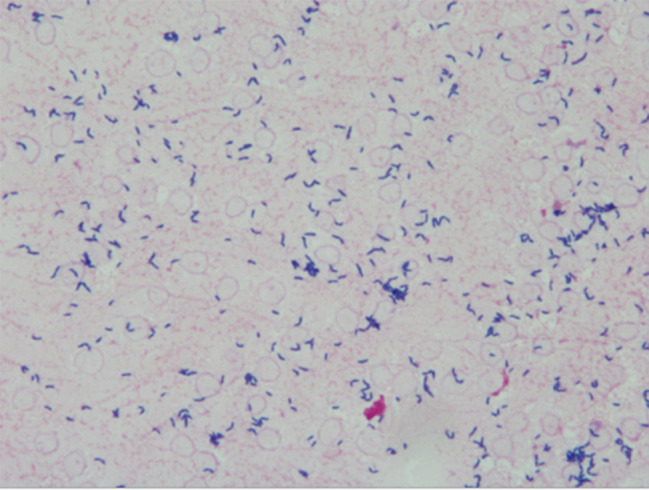
Gram stain findings of organism detected in two sets of blood cultures. Gram staining of the blood culture isolates revealed gram-positive rods. The bacterial cells appeared as pleomorphic rods with some curved or club-shaped forms. The staining was performed using the standard Gram staining method, and the microscopic observation was conducted at a magnification of 1000×

On day 10, he had a fever of 38.1 °C, and after two sets of blood cultures were taken, 3 g of sulbactam/ampicillin (SBT/ABPC) was administered twice a day (Fig. 1). Blood tests showed a mildly elevated CRP level of 5.61 mg/dL, and urinalysis and chest X-ray showed no evidence of urinary tract infection or pneumonia. Two sets of blood cultures were negative. He was diagnosed with mild bronchitis and was treated until day 17. On day 18, he had a fever of 39.5 °C, blood pressure decreased to 81/38 mmHg, pulse rate increased to 127 bpm, and shivering was struck him. After blood tests and 2 sets of blood cultures were taken, SBT/ABPC was continued. Blood tests revealed an increase in WBC to 10,280/µL and an increase in CRP to 1.61 mg/dL, chest X-ray indicated mild pneumonia, and urinalysis showed no evidence of urinary tract infection. On day 19, the bacteriology laboratory reported the development of gram-positive rods from two sets of blood cultures (Fig. 2). Blood cultures were positive approximately 12–14 h after initiation, the first in aerobic and anaerobic bottles and the second in aerobic bottles only. He became delirious again and self-extracted on a peripheral infusion. His peripheral venous puncture site of right arm was found to be infected, a catheter-related bloodstream infection (CRBSI) caused by *Corynebacterium* spp. was assumed. The peripheral infusion was replaced on the left arm. The infusion was changed again to a 12-hour infusion within daytime due to the risk of self-extraction. From these things, treatment using vancomycin (VCM) was initiated. Initial dose of VCM was 1,000 mg (27.4 mg/kg) and maintenance dose of VCM was 500 mg (13.7 mg/kg) every 12 h, administered via intravenous infusion over one hour. His estimated weight at this time was 36.5 kg, his creatinine level was 0.54 mg/dL, and his creatinine clearance, calculated using the Cockcroft-Gault equation, was 47.9 mL/min. On day 22, the trough and peak blood concentrations of VCM were 11.4 µg/mL and 18.4 µg/mL, respectively. From the blood concentrations obtained, the steady-state AUC was estimated to be 356.3 µg·h/mL using Bayesian estimation software PAT (Practical AUC-Guided TDM for Vancomycin). [10]. To date, the organism had not yet been identified, but the MIC_50_ for VCM was determined to be 2 µg/mL by broth microdilution method Table 1. The identification of the organism using a commercial identification kit, API Coryne^®^ (bioMérieux Marcy-l’Étoile, France), was unsuccessful. Therefore, species-level identification was outsourced to an external laboratory. After the start of VCM, the temperature remained at approximately 37 °C, WBC decreased (from 10,280 to 6,930 /µL) and there was no worsening of oxygenation, but an increase in CRP (from 1.61 to 3.61 mg/dL) was observed. In PAT, an AUC of 445 µg·h/mL was expected to be reached by increasing the dose to 1,250 mg once daily. Therefore, the dose of VCM was increased to 1250 mg once daily to achieve an AUC of 400 µg·h/mL or higher, in accordance with the AUC target value for MRSA infection. On day 25, MALDI-TOF-MS analysis at a laboratory identified the detected organism as *Arthrobacter woluwensis*. This analysis was conducted using Bruker MALDI Biotyper System^®^ (Bruker Corporation, Billerica, MA, USA) by an external laboratory. This analysis was conducted in accordance with the CLSI M100 32nd edition (2022). On the same day, the trough and peak blood concentrations of VCM were 13.4 µg/mL and 32.7 µg/mL respectively, an AUC was estimated to be 471.9 µg·h/mL by Bayesian estimation with PAT, so VCM was continued at the same dose. VCM was administered for 14 days. During the course of the treatment, there was no worsening of clinical condition, and no adverse events occurred such as red-neck syndrome or renal dysfunction. On day 30, two sets of blood cultures were taken to confirm the absence of bacteremia, and by day 37 there was no growth. After treatment, his BT remained approximately 37 °C, and his WBC and CRP level remained at approximately 9,000/µL and 2 mg/dL respectively, but there was no worsening of his general condition. On day 40, transthoracic echocardiography was performed, and there was no indication of infective endocarditis. For the record, his medical history, anamnesis and imaging findings confirmed that he had no medical devices implanted in his body, including a heart. On day 43, no self-extraction occurred since day 20, he was transferred to a convalescent hospital.

**Table 1:** Antimicrobial susceptibility of *Arthrobacter woluwensis* isolated from two sets of blood culture

Antibiotic	MIC_50_(µg/mL)	Antibiotic	MIC_50_(µg/mL)
PCG	2	MINO	< 1
ABPC	4	EM	≦ 0.25
CVA/AMPC	2	CAM	≦ 0.25
SBT/ABPC	> 4	AZM	≦ 0.12
CEZ	> 2	CLDM	1
CFDN	> 1	LVFX	4
CTX	> 2	MFLX	1
CTRX	> 2	VCM	2
CFPM	> 2	ST	≦ 0.5
IPM/CS	0.5		
MEPM	> 2		

PCG, penicillin G; ABPC, ampicillin; CVA/AMPC, clavulanic acid/amoxicillin; CEZ, cefazolin; CFDN, cefdinir; CTX, cefotaxime; CTRX, ceftriaxone; CFPM, cefepime; IPM/CS, imipenem/cilastatin; MEPM, meropenem; MINO, minocycline; EM, erythromycin; CAM, clarithromycin; AZM, azithromycin; CLDM, clindamycin; LVFX, levofloxacin; MFLX, moxifloxacin; ST, sulfamethoxazole/trimethoprim;

## Discussion and conclusions

*Arthrobacter* spp. are endemic to natural environments, such as soil [1], and are generally considered to have low pathogenic potential and no standard treatment has been established. There have been 7 reported cases of bacteremia by *Arthrobacter woluwensis* Table 2, [2–8]. *Arthrobacter* spp. is a rare organism, and its identification using commercially available kits such as API Coryne may be challenging. Previous reports have identified *Arthrobacte*r spp. using genetic identification methods such as 16 S rRNA sequencing [2–8] or mass spectrometry [1]. In our case, API Coryne failed to provide a definitive identification, necessitating further analysis through MALDI-TOF MS at an external laboratory. This suggests that routine clinical identification methods may not always be sufficient for detecting *Arthrobacter spp.*, highlighting the need for advanced identification techniques in certain cases. Consequently, infections caused by *Arthrobacter* spp. may be underreported because they have been treated as *Corynebacterium* spp. infection in general clinical practice. Cases of previous reports include immunosuppressed states due to malignancy [4, 6, 7], regular use of intravenous drugs [3, 8], and conditions involving skin barrier disruption such as indwelling intravenous catheters [2, 4, 6]. The two cases [5, 7] may also have been treated with intravenous injections due to their underlying disease. In our case, the patient was immunocompromised due to esophageal cancer and poor nutrition, as well as the possibility of bacterial invasion and infection due to daily puncture of the intravenous line. These findings suggest that immunosuppressed conditions and the disruption of skin and vessel walls by intravenous injection and catheter placement are important pathways of invasion by *A. woluwensis*. In these reports, *A.woluwensis* has shown high MIC_50_ for penicillins, cephalosporins, and fluoroquinolones, and relatively low MIC_50_ for anti-MRSA drugs such as vancomycin, teicoplanin, and linezolid. With the exception of one case [8], all cases were treated with antimicrobial agents based on susceptibility. And with the exception of one discharged case [4], all cases were cured. In our case, blood culture confirmed gram-positive rod, and VCM was administered as an empirical treatment, assuming *Corynebacterium* spp. bacteremia. The organisms detected were susceptible to penicillins, Imipenem/Cilastatin, and fluoroquinolones as well as VCM Table 1. However, because oral administration was difficult due to esophageal cancer, and his infusion line was frequently self-extracted due to delirium, penicillins which require frequent administration, could not be selected. In addition, to avoid the occurrence of antimicrobial-resistant bacteria and *Clostridioides difficile* infection, broad-spectrum antimicrobial agents, such as carbapenems and fluoroquinolones, were withheld, resulting in the continuation of administration of VCM.

**Table 2:** Previous reported cases of *Arthrobacter woluwensis* bacteremia

Reported year	Age	Sex	Underlying condition	Diagnosis	Antibiotics treatment	MIC_50_ for VCM (µg/mL)	Duration of therapy	Outcome
1996 [2]	33	F	Human immunodeficiency virus infection Venous port	Venous port infection	ABPC	2	14	Cured
2004 [3]	39	M	Intravenous drug user Tuberculosis infection	Infective endocarditis	TEIC	2	42	Cured
2006 [4]	56	M	Colorectal cancer Subclavian catheter	Bacteremia Subclavian catheter infection	VCM	1.5	14	Discharged
2007 [5]	91	F	Ischemic stroke	Bacteremia	LZD	2	10	Cured
2012 [6]	76	F	Multiple myeloma Central venous port	Bacteremia Central venous port infection	TEIC	2	19	Cured
2021 [7]	93	M	Prostate cancer Stomach cancer Coronary artery disease	Bacteremia	ABPC	1	14	Cured
2021 [8]	52	ND	Intravenous drug user Hepatitis C	Infective endocarditis	DAP + ST	ND	ND	Cured
Present case	89	M	Stomach ulcer Esophageal cancer Intravenous catheter	Bacteremia Peripheral catheter infection	VCM	2	14	Cured

ABPC, ampicillin; DAP, daptomycin; ST, sulfamethoxazole/trimethoprim; TEIC, teicoplanin; ND, no data;

When administering VCM, it is recommended that the dosing design be based on the AUC/MIC to enhance clinical efficacy and reduce the incidence of renal dysfunction [9]. Therefore, TDM was performed to assess the AUC in our case. Blood concentrations were measured 1 h after the end of VCM administration (peak) and before administration (trough), and the AUC was calculated by simulating blood concentrations through Bayesian estimation using TDM analysis support software [10]. The most common MIC_50_ for VCM in previous reports of *A.woluwensis* bacteremia is 2 µg/mL. Although cases of treatment with VCM have been reported, there are no data of dosages, blood concentrations, and TDM. In our case, the MIC_50_ for VCM for *A. woluwensis* was also 2 µg/mL, but clinical improvement was observed when VCM was used at an AUC of ≥ 400 µg·h/mL as an indicator. When MIC_50_ of MRSA is 2 µg/mL, it is theoretically difficult to design a dosing regimen with an AUC/MIC of 400–600. Japanese guidelines recommend an AUC/MIC of 400 or more, irrespective of the MIC_50_ [9]. In *Staphylococcus aureus* bacteremia, meta-analyses have reported no increased mortality in the high MIC group (MIC ≥ 1.5 µg/mL) compared with the low MIC group [11]. On the other hand, some reports suggest that vancomycin is less effective in the high MIC group [12, 13], requiring a clinical decisions to be made, such as continued administration or switching to another agent. About the therapeutic efficacy of vancomycin in non-MRSA infections, efficacy has been demonstrated in *Enterococcus faecium* bacteremia with AUC/MIC ≥ 414 [14], and AUC/MIC ≥ 389 [15]. However, poor prognosis has been reported in *Enterococcus faecium* bacteremia at MIC ≥ 1 µg/mL [16], and caution should be exercised with MIC values. About other gram-positive cocci such as *Staphylococcus epidermidis* and *Streptococcus pneumoniae*, the relationship between the AUC and MIC of vancomycin and its clinical efficacy has not been well studied. For gram-positive rods such as *Corynebacterium* spp. (e.g., *Corynebacterium striatum* [17] and *Corynebacterium urealyticum* [18], treatment with vancomycin is recommended, but clinically effective blood concentrations have not been studied. According to the CLSI breakpoint criteria, the susceptibility of *Arthrobacter* spp. to vancomycin is considered to be ≤ 2 µg/mL, with reference to the breakpoint for *Corynebacterium* spp. As bacterial identification techniques advance and become more widespread, previously rare gram-positive rods, such as *Arthrobacter spp.*, may be more frequently identified in clinical settings. Consequently, the relationship between AUC of vancomycin and the MIC_50_ of organisms, particularly when it is 2 µg/mL, will become an increasingly important issue that requires further investigation. Our case suggests that vancomycin treatment with a target AUC of ≥ 400 µg·h/mL is acceptable in bacteremia caused by *A. woluwensis*, even at an MIC_50_ of 2 µg/mL. However, since this conclusion is based on a single case, further studies are needed to validate its effectiveness. Additionally, a limitation of this discussion is that catheter replacement may have contributed to the treatment outcome, as the infection was suspected to be catheter-related bloodstream infection. In previous reports of catheter-related bloodstream infections [2, 4, 6], not only antimicrobial treatment but also catheter removal at the time of suspected infection was used, which subsequently led to cure. Therefore, in addition to vancomycin therapy, catheter removal should also be considered to have an important role in treatment.

The duration of therapy was completed after 14 days of administration, although it is possible that a shorter period could have been completed due to the patient’s condition and the effects of catheter removal. However, most treatment durations of the other cases caused by *A. woluwensis* bacteremia have been at least 14 days, and a 14-day duration may be acceptable if the patient is in good condition and the source of infection is under control.

After treatment, the patient was in good condition, blood cultures became negative, and there were no new findings of CRBSI or infective endocarditis. The appearance of inflammatory reactions and low-grade fever persisted but was thought to be due to malignancy, and no additional imaging or other tests were performed. Therefore, infections of other organs and infections due to other pathogens could not be ruled out.

In conclusion, this is the first report showing that VCM with TDM is a promising treatment for *A. woluwensis* bacteremia and could provide useful information for appropriate treatment. However, as mentioned earlier, there have been few reports on this topic, further studies are needed to determine the optimal treatment method.

## Data Availability

No datasets were generated or analysed during the current study.

## Abbreviations

AUC: Area under the concentration-time curve
BT: Body temperature
CRBSI: Catheter-related blood stream infection
CRP: C-reactive protein
MALDI-TOF MS: Matrix Assisted Laser Desorption/Ionization Time-of-flight mass spectrometry
MIC_50_: Minimum inhibitory concentration for 50% of isolates
MRSA: Methicillin-resistant Staphylococcus aureus
SBT/ABPC: Sulbactam/ampicillin
TDM: Therapeutic drug monitoring
VCM: Vancomycin
WBC: White blood cell
